# Supplementary material for: Alternative Splicing of Pericentrin Contributes to Cell Cycle Control in Cardiomyocytes
Source: J Cardiovasc Dev Dis. 2021 Jul 27;8(8):87. doi: 10.3390/jcdd8080087 (PMC8397033; doi:10.3390/jcdd8080087)
Supplement: Supplementary file 1 [file jcdd-08-00087-s001.zip › jcdd-1209136-supplementary.pdf]

Supplementary Material

Alternative splicing of pericentrin contributes to cell cycle control in cardiomyocytes

Jakob Steinfeldt, Robert Becker, Silvia Vergarajauregui, and Felix B. Engel

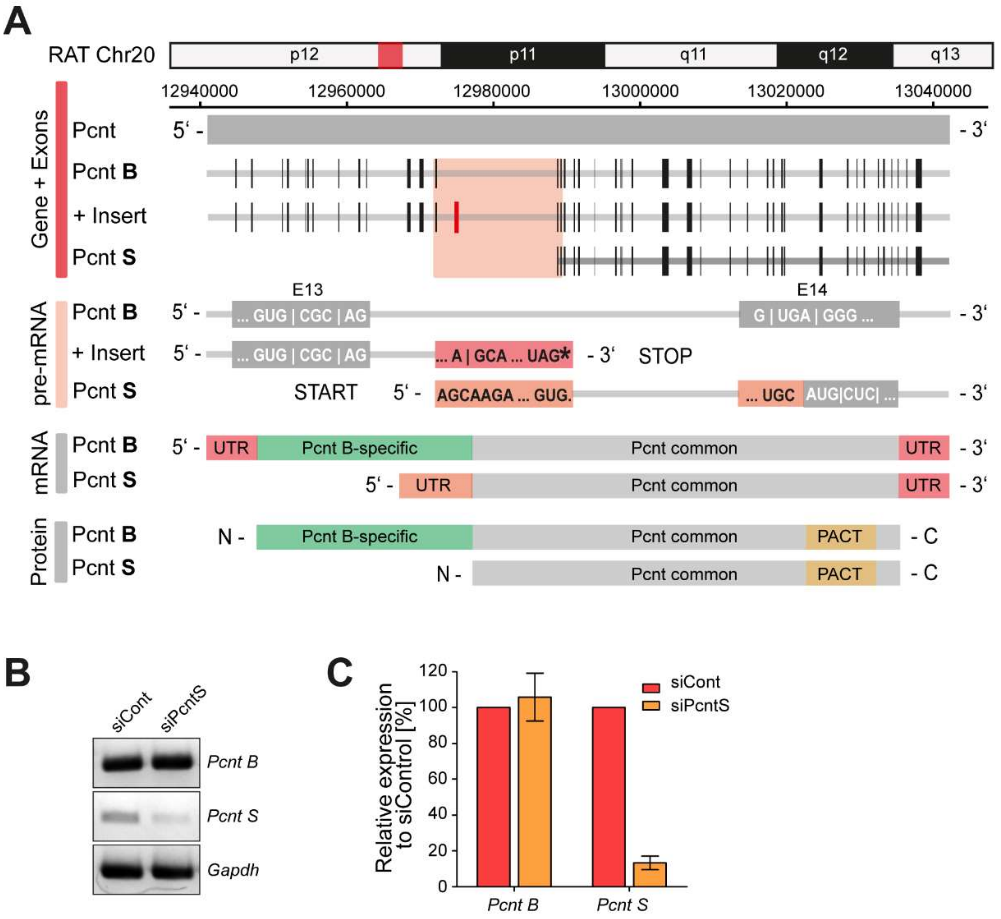

Figure S1. Overview of alternative splicing of PCNT and challenges for specific depletion of Pcnt S. (**A**) Alternative splicing of rat *Pcnt* results in the insertion of an intronic region in the *Pcnt* gene between exon 13 and 14, which becomes the first exon encoding the isoform Pcnt S (Gene + Exon). This exon contains a stop codon and consequently initiation of *Pcnt S* translation occurs in a downstream start codon resulting in the truncation of the first 232 amino acids at the N-terminal region of the longer Pcnt B isoform. The new 5'-UTR is constituted of the sequence between stop codon and new start codon, in part consisting of a coding sequence for *Pcnt B* (pre-mRNA). The lack of specific regions in the expression of *Pcnt S* presents a major challenge for specific Pcnt S depletion and precludes Pcnt S-specific antibody staining. To specifically deplete Pcnt S we used a siRNA against the specific part of the 5'-UTR of the *Pcnt S* mRNA (mRNA). (**B**) RT-PCR of *PcntB*, *PcntS* and *Gapdh* utilizing RNA from P3 cardiomyocytes transfected with the corresponding siRNA as indicated. (**C**) Quantification of B, as *Pcnt* band intensity normalized to *Gapdh* band intensity.

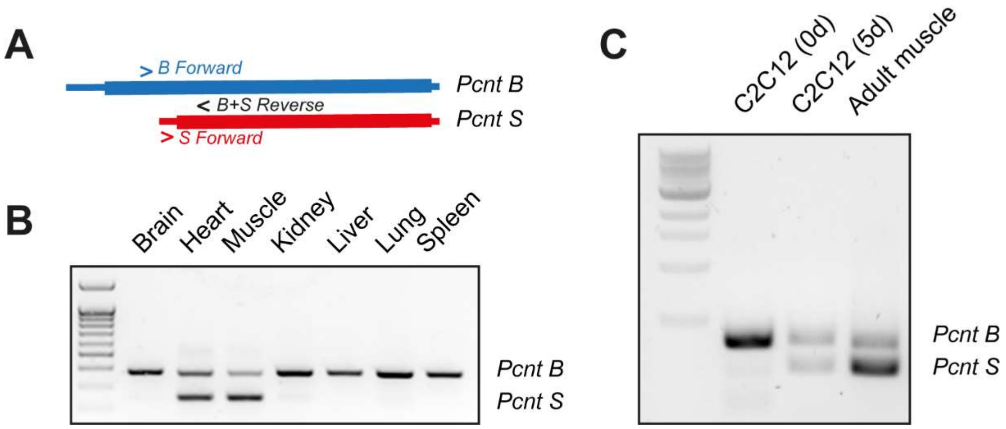

Figure S2. RT-PCR using three primers to detect *Pcnt B* and *Pcnt S*. (**A**) Scheme of the primers used. Forward-primer specific for *Pcnt B* is shown in blue, forward-primer specific for *Pcnt S* in red, and the reverse primer detecting both isoforms is shown in black. (**B**,**C**) Three primer RT-PCR from RNA isolated from the indicated adult mouse tissues (B) or from C2C12 at myoblast stage (0d), or after 5 days of differentiation (5d) in comparison to adult muscle tissue (C).

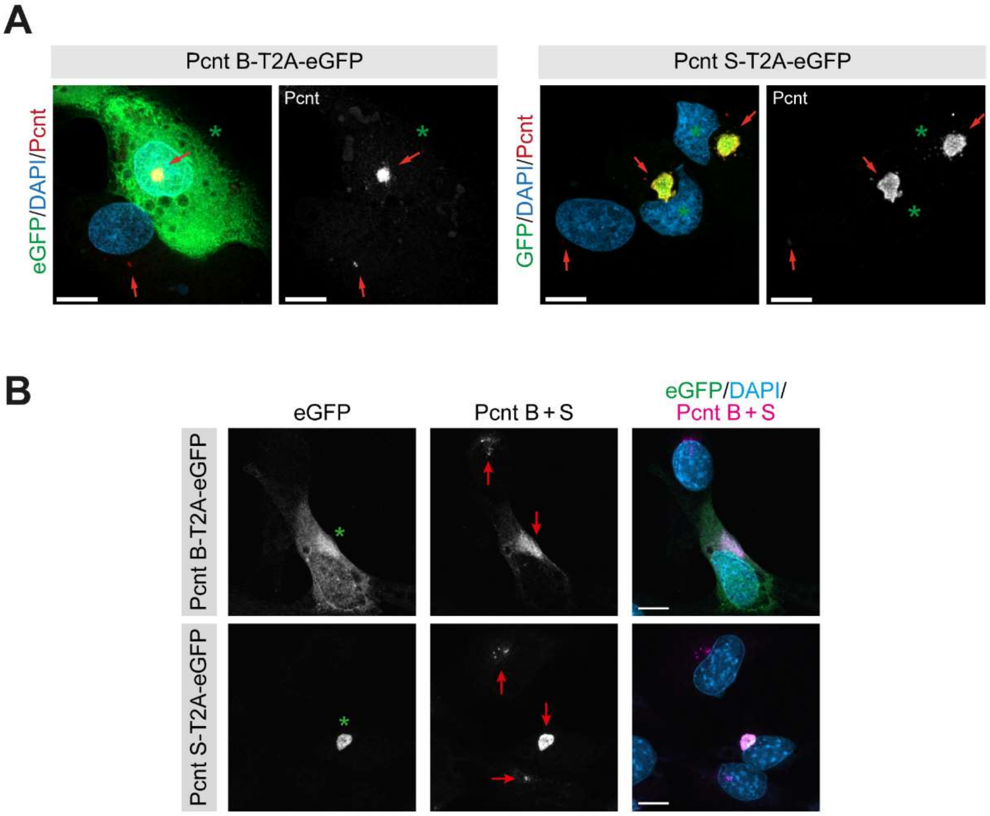

Figure S3. Verification of Pcnt expression after ectopic expression in ARPE-19 adult retinal pigment epithelial cells and C2C12 myoblasts. Representative examples of ARPE-19 cells (A) or C2C12 myoblasts (B) transfected with bi-cistronic plasmids encoding Pcnt B-T2A-eGFP or Pcnt S-T2A-eGFP (GFP, green) and immunostained with anti-Pcnt antibody (red). Nuclei were visualized with DAPI (DNA, blue). Green asterisk: GFP+ cell; Red arrow: Pcnt staining. Scale bars: 10 μm.

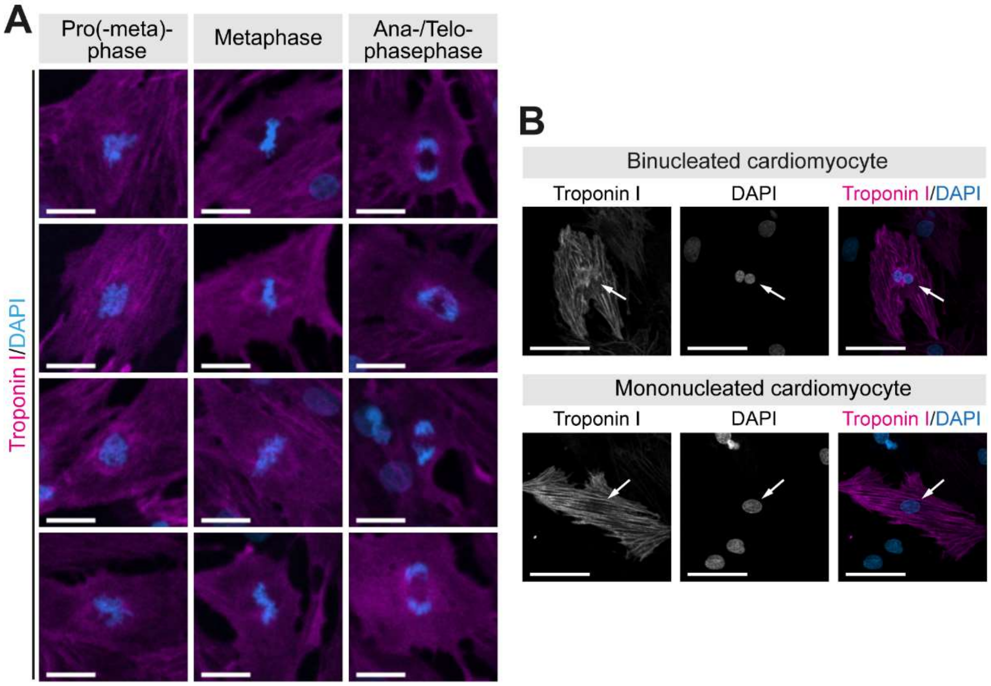

Figure S4. Representative examples of different cell cycle stages of cardiomyocytes. (**A**) Representative examples of cardiomyocytes (troponin I) in pro(-meta)phase, metaphase and ana-/telophase. Chromosomes were visualized with DAPI (DNA). Scale bars: 20 μm. (**B**) Representative examples of mono- and binucleated cardiomyocytes (troponin I). Nuclei were visualized with DAPI (DNA). White arrows: cardiomyocyte nuclei. Scale bars: 50 μm.
